# Prognostic Value of Non-nodal Regional Metastases in Predicting Sentinel Lymph Node Status in Cutaneous Melanoma: Multicenter Analysis of the Sentinel Lymph Node Working Group Database

**DOI:** 10.1245/s10434-026-19086-2

**Published:** 2026-01-23

**Authors:** Nazia Riaz, Stanley P. Leong, Mohammed Kashani-Sabet, Richard L. White, John T. Vetto, Schlomo Schneebaum, Cristina O’Donoghue, J. Harrison Howard, Eli Avisar, Jukes P. Namm, Heidi Kosiorek, Barbara Pockaj, Mark Faries, Giorgos Karakousis, Jonathan S. Zager, Roger Olofsson Bagge

**Affiliations:** 1 Department of Surgery, Institute of Clinical Sciences, Sahlgrenska Academy, University of Gothenburg, Gothenburg, Sweden; 2 Department of Surgery, Sahlgrenska University Hospital, Gothenburg, Västra Götalandsregionen Sweden; 3 Center for Melanoma Research and Treatment, California Pacific Medical Center and Research Institute, San Francisco, CA USA; 4 Department of Surgery, Atrium Health Levine Cancer Center, Charlotte, NC USA; 5 Department of Surgery and Division of Surgical Oncology, Oregon Health & Science University, Portland, OR USA; 6 Department of Surgery, Tel Aviv University, Tel Aviv, Israel; 7 Department of Surgery, Rush University Medical Center, Chicago, IL USA; 8 Department of Surgery, University of South Alabama, Mobile, AL USA; 9 Department of Surgical Oncology, University of Miami School of Medicine, Miami, FL USA; 10 Department of Surgery, Loma Linda University, Loma Linda, CA USA; 11 Department of Quantitative Health Sciences, Mayo Clinic, Scottsdale, AR USA; 12 Department of General Surgery, Division of Surgical Oncology, Mayo Clinic Arizona, Phoenix, AR USA; 13 Department of Surgery, Angeles Clinic and Research Institute, Cedars-Sinai Medical Center, Los Angeles, CA USA; 14 Division of Endocrine and Oncologic Surgery, University of Pennsylvania School of Medicine, Philadelphia, PA USA; 15 Departments of Cutaneous Oncology and Sarcoma, Moffit Cancer Center, Tampa, FL USA

## Abstract

**Background:**

Non-nodal regional metastases, including microsatellite lesions, satellites, and in-transit metastases, are an uncommon but aggressive entity in cutaneous melanoma. We evaluated their association with clinicopathological features, sentinel lymph node (SLN) status, and prognosis.

**Patients and Methods:**

The Sentinel Lymph Node Working Group (SLNWG) database was used to examine the clinicopathological associations of non-nodal regional metastases, their prognostic significance in relation to SLN status, and their impact on clinical outcomes, including in patients with negative SLN status, using multivariable logistic regression and Cox regression models, respectively.

**Results:**

Of 13,474 patients in the SLNWG database, 12,644 underwent SLN biopsy, and 3.4% (*n* = 426) had non-nodal regional metastases at diagnosis. These were associated with adverse clinicopathological features, higher odds of SLN positivity (OR 2.75; 95% CI 2.18–3.47; *P* < 0.001), and worse relapse-free survival (RFS: HR 1.47, 95% CI 1.07–2.02, *P* = 0.02), melanoma specific survival (MSS: HR 1.72, 95% CI 1.06–2.78, *P* = 0.03), overall survival (OS: HR 1.49, 95% CI 1.01–2.21, *P* = 0.05). Adverse prognostic associations were also observed in the SLN negative subgroup (RFS: HR 1.93; 95% CI 1.58–2.36, *P* < 0.001; MSS: HR 2.24; 95% CI 1.58–3.17, *P* < 0.001, and OS: HR 1.51; 95% CI 1.17–1.95, *P* = 0.002).

**Conclusions:**

Non-nodal regional metastases independently predict SLN involvement and adverse prognosis, and outcomes remain poor even when SLN is negative. These findings reinforce the risk stratification and prognostic relevance of SLN assessment in clinically node-negative patients with non-nodal regional metastases.

**Supplementary Information:**

The online version contains supplementary material available at 10.1245/s10434-026-19086-2.

Loco-regional metastases, including microsatellites, satellites, and in-transit metastases, represent a heterogeneous spectrum of non-nodal regional metastases that are peculiar to cutaneous melanoma.^1^ The development of non-nodal, regional nodal, and distant metastases has been linked with distinct metastatic pathways, including extravascular migratory metastasis, whereby angiotropic melanoma cells at the advancing edge associate with the abluminal surface of blood vessels. Uniquely, the process involves pericytic mimicry, in which melanoma cells replace the native pericytes and spread along the vascular channels at a distance from the primary lesion.^2^

Microsatellites are traditionally identified on histopathological examination as cutaneous or subcutaneous metastatic lesions located adjacent to or beneath a primary cutaneous melanoma, characteristically separated from it by intervening benign stroma. In contrast, the other two forms, satellite lesions and in-transit metastases, are clinically apparent cutaneous or subcutaneous metastases occurring between the primary tumor or the wide local excision scar and the regional lymph nodes but not in direct continuity with the primary lesion. When these metastatic foci are within 2 cm of the primary melanoma, they are referred to as satellite lesions; if located more than 2 cm away, they are termed in-transit metastases.^1^

Although rare, the presence of non-nodal regional metastases at diagnosis has been associated with aggressive clinicopathological features,^3–5^ higher frequency of sentinel lymph node (SLN) positivity, and adverse prognosis.^4–10^ When present, non-nodal regional metastases meet the criteria for at least stage IIIB disease and assign the nodal “N” category to “c” subcategories (i.e., N1c, N2c, and N3c) depending on the absence (N1c) or presence (N2c–N3c) of regional lymph node involvement.^1^ Even though these patients already have stage III disease, SLN status remains prognostically important: the 5 year melanoma-specific survival (MSS) ranges from ~93% in stage IIIA to ~32% in stage IIID disease. Therefore, performing SLN biopsy in this setting offers incremental risk prognostication in the clinically node-negative subgroup of patients with non-nodal regional metastases.^11–13^

Sentinel lymph node biopsy is a recognized standard in melanoma staging and prognosis. It enhances staging precision and strengthens prognostic predictions when used in conjunction with primary tumor characteristics.^1,14,15^ However, the independent prognostic value of non-nodal regional metastases in relation to SLN status has been examined in only a limited number of studies, likely due to the rarity of this entity.^4,9,16^

In this study, we leveraged the robust multi-institutional Sentinel Lymph Node Working Group (SLNWG) database to investigate the association of non-nodal regional metastases with clinicopathological features, the prognostic significance of non-nodal regional metastases in informing SLN status, and their impact on clinical outcomes. Furthermore, we also examine the prognostic relevance of non-nodal regional metastases in patients with negative SLN status.

## Patients and Methods

### Study Population

The SLNWG cohort characteristics have been described in detail previously.^17^ Briefly, the current retrospective investigation included all adult patients who underwent SLN biopsy between 1988 and 2025, for whom detailed clinicopathological data and follow-up information on oncological outcomes are available within the SLNWG database. Patients with stage IV disease were excluded from the study. All centers involved adhered to the respective institutional or national standards for diagnostic excisional biopsies, wide local excisions, and SLN biopsy, pathological evaluation of the specimens, and documentation of outcomes. Clinicopathological data from all participating centers, including histopathological features of microsatellites, satellites, and in-transit metastases, were submitted to the SLNWG database; no additional pathology review was undertaken for this study. Non-nodal regional metastases were reported by the center pathologists according to the American Joint Committee on Cancer (AJCC) criteria in use at the time of diagnosis, as documented in the original pathology reports.^1^ In line with AJCC classification, for the current study, microsatellites, satellites, and in-transit metastases were grouped under the umbrella term of “non-nodal regional metastases.” The SLNWG database records the presence of microsatellites, satellites, and in-transit metastases but does not specify whether these were identified clinically prior to SLN biopsy or pathologically on the initial diagnostic biopsy or subsequent WLE, reflecting variability in staging and documentation across contributing institutions. Information regarding completion of lymph node dissection or nodal surveillance after a positive SLN was not included in the analysis, as these data are not consistently recorded in the SLNWG database. Race and ethnicity data were not uniformly recorded, and hence are not presented in the current study.

The study was performed in line with the Strengthening the Reporting of Observational Studies in Epidemiology (STROBE) guidelines.^18^ Each SLNWG member obtained institutional ethical approval for the use of deidentified research data.

### Statistical Analyses

The associations between clinicopathological features and presence or absence of non-nodal regional metastases were estimated by chi-squared test, and corresponding frequencies and percentages are reported.

Multivariable logistic regression analysis was performed to identify factors associated with SLN positivity. Independent variables in the model included age, sex, anatomical sites of involvement, histological subtype of melanoma, ulceration, Breslow thickness, vascular invasion, regression status, mitotic rate, and non-nodal regional metastases. Age and Breslow thickness were used as continuous variables, and the others as categorical. Missing data were handled using multiple imputation by chained equations (MICE) under the assumption of missing data at random (MAR). A total of 20 imputed datasets were generated using predictive mean matching for continuous variables, logistic regression for binary variables, and multinomial logistic regression for categorical predictors with > 2 levels. The outcome (SLN status) was not imputed. Multivariable logistic regression models were fitted within each imputed dataset, and parameter estimates were combined using Rubin’s rules to derive pooled odds ratios (OR) with 95% confidence intervals (CIs). All prespecified variables were entered simultaneously into the model.

Relapse-free survival (RFS) was defined as the time from diagnosis to the first melanoma recurrence or death due to any cause. Melanoma-specific survival (MSS) was defined as the time from diagnosis to death attributable to melanoma, with deaths from other causes censored at the date of the death, while overall survival (OS) was defined as the time from diagnosis to death from any cause.

Competing risks analysis was used to estimate the cumulative incidence of local, regional, and distant relapse, with other relapse types and death without relapse as competing events. Cumulative incidence functions (CIFs) were estimated using the Fine–Gray method, and differences between groups were assessed with the subdistribution hazard models. Non-nodal regional metastases were included as the main covariate of interest. Univariate associations with the study endpoints were examined using Cox proportional hazards regression, and survival distributions were illustrated with Kaplan–Meier curves and numbers-at-risk tables, which are displayed up to 10 years. For univariable analyses, events occurring after 10 years are treated as censored. Multivariable Cox regression models were used to identify factors that independently influence prognosis. Candidate variables included age, sex, anatomical site, histological subtype, ulceration, Breslow thickness, vascular invasion, regression status, and SLN status. Missing data were handled using MICE as described above (20 imputations); survival outcomes (time-to-event and censoring) were not imputed. Cox models were fitted within each imputed dataset, and pooled hazard ratios (HRs) with 95% CIs were obtained using Rubin’s rules. The proportional hazards assumption was evaluated using scaled Schoenfeld residuals. An initial violation for mitotic rate led to its inclusion as a stratification factor; no other variables showed evidence of non-proportional hazards, and the global Schoenfeld test after stratification was nonsignificant (*P* = 0.46). Statistical analyses were performed using RStudio (Posit software, version 2025.05.1+513) and SPSS Statistics (IBM version 29.0.2.0).

## Results

### Clinicopathological Factors Associated with Non-nodal Regional Metastasis

The SLNWG registry comprises 13,474 patients who were diagnosed with cutaneous melanoma between 1988 and 2025. The median age of the patients was 62 years (range 49–72 years), of whom 58% (*n* = 7801) were male. Most patients were diagnosed with stage I (45.3%, *n* = 6748), followed by stage II (30.4%, *n* = 4530), stage III (14.7%, *n* = 2128), and stage IV (1.4%, *n* = 204). Sentinel lymph node biopsy was performed in 12,644 patients, of whom 14.6% (*n* = 1973) patients had SLN metastasis. Among the patients subjected to SLN biopsy, non-nodal regional metastases were identified in 426 patients at diagnosis. The predominant form of non-nodal regional metastasis was microsatellite lesions (2.4%, *n* = 323), followed by satellite nodules (0.5%, *n* = 71) and in-transit metastases (0.2%, *n* = 32). The median duration of follow-up was 3.2 years (range 1.0–7.3 years). Among all the patients who underwent SLN biopsy, local, regional, and distant recurrences were documented in 2.9% (*n* = 372), 6.8% (*n* = 857), and 7.5% (*n* = 946) patients, respectively. The clinicopathological features are summarized in Table 1. Non-nodal regional metastasis showed a significant correlation with adverse clinicopathological features, including age ≥ 62 years, positive margin status, trunk as the primary melanoma site, increasing Breslow thickness, higher Clark level, mitotic rate, ulceration, vascular invasion, and SLN metastases (*P* < 0.05).

**Table 1:** Clinicopathological features of patients with and without non-nodal regional metastasis

Clinicopathological features	Non-nodal regional metastases		*P*-value
	Absent (*n* = 13,048)	Present (*n* = 426)	
*Sex*			
Male	7540 (57.8)	261 (61.3)	0.34
Female	5505 (42.2)	165 (38.7)	
Missing	3 (0.0)	0 (0.0)	
*Age (years)*			
< 62	6504 (49.8)	176 (41.3)	< 0.001
≥ 62	6544 (50.2)	250 (58.7)	
Missing	0 (0.0)	0 (0.0)	
Margin status*			
Negative	4234 (32.4)	88 (20.7)	< 0.001
Positive	5491 (42.1)	229 (53.8)	
Missing	3323 (25.5)	109 (25.6)	
*Primary region*			
Head and neck	2427 (18.6)	102 (23.9)	0.005
Upper extremity	2966 (22.7)	86 (20.2)	
Lower extremity	2872 (22)	109 (25.6)	
Trunk	4767 (36.5)	129 (30.3)	
Missing	16 (0.1)	0 (0.0)	
*Breslow thickness (mm)*			
< 1.0	3613 (27.7)	39 (9.2)	< 0.001
1.01–2.00	4682 (35.9)	72 (16.9)	
2.01–4.00	2663 (20.4)	133 (31.2)	
> 4.00	2039 (15.6)	178 (41.8)	
Missing	51 (0.4)	4 (0.9)	
*Clark level*			
I	3 (0)	0 (0)	< 0.001
II	528 (4.0)	3 (0.7)	
III	2711 (20.8)	38 (8.9)	
IV	5797 (44.4)	185 (43.4)	
V	612 (4.7)	78 (18.3)	
Missing	3397 (26)	122 (28.6)	
*Ulceration*			
Absent	9129 (70.0)	218 (51.2)	< 0.001
Present	3572 (27.4)	199 (46.7)	
Missing	347 (2.7)	9 (2.1)	
*Vascular invasion*			
Absent	8645 (66.3)	229 (53.8)	< 0.001
Present	558 (4.3)	101 (23.7)	
Missing	3845 (29.5)	96 (22.5)	
*Mitotic rate*			
< 1/mm^2^	1409 (10.8)	21 (4.9)	< 0.001
≥ 1/mm^2^	7010 (53.7)	277 (65)	
Missing	4629 (35.5)	128 (30)	
*Regression rate*			
Absent	8118 (62.2)	261 (61.3)	0.43
Present	1504 (11.5)	43 (10.1)	
Missing	3426 (26.3)	122 (28.6)	
*Melanoma type*			
Superficial spreading	4070 (31.2)	99 (23.2)	< 0.001
Nodular	2336 (17.9)	120 (28.2)	
Acral lentiginous	303 (2.3)	16 (3.8)	
Lentigo maligna	350 (2.7)	15 (3.5)	
Others	2600 (19.9)	72 (16.9)	
Missing	3389 (26)	104 (24.4)	
*Tumor infiltrating lymphocytes*			
Non-brisk	3481 (26.7)	107 (25.1)	0.003
Brisk	1102 (8.4)	18 (4.2)	
Missing	8465 (64.9)	301 (70.7)	
*Sentinel lymph node status*			
Negative	10,444 (80)	227 (53.3)	< 0.001
Positive	1799 (13.8)	174 (40.8)	
Missing	805 (6.2)	25 (5.9)	

*Margin status indicates the margin status of the initial diagnostic biopsy (shave or excision)

### Clinicopathological Factors Associated with Sentinel Lymph Node Status in the SLNWG Cohort

**Fig. 1:**
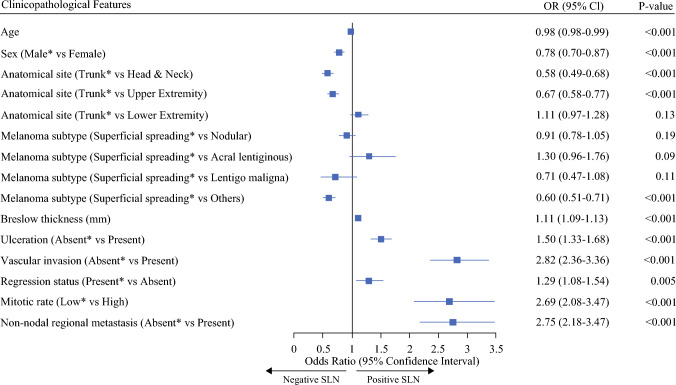
Multivariable logistic regression analysis (multiple imputations, *n* = 20) showing OR with 95% CI and corresponding *P*-values for independent association of clinicopathological features with the SLN status in the SLNWG cohort (*n* = 12,644); age and Breslow thickness are used as continuous variables; non-nodal regional metastases include de novo microsatellites, satellites, and in-transit metastases. *Indicates the reference categories; *OR* odds ratio, *CI* confidence intervals, *SLN* sentinel lymph node

We performed multivariable logistic regression to determine the independent prognostic significance of clinicopathological factors influencing SLN status in the study cohort. Model discrimination was acceptable (mean area under the curve, AUC 0.73). Consistent with previous reports,^4,9,16^ the presence of non-nodal regional metastases was independently associated with a nearly threefold higher odds of SLN positivity (OR 2.75, 95% CI 2.18–3.47, *P* < 0.001) (Fig. 1).

### Prognostic Value of Sentinel Lymph Node Status in Patients with Non-nodal Regional Metastases

**Fig. 2:**
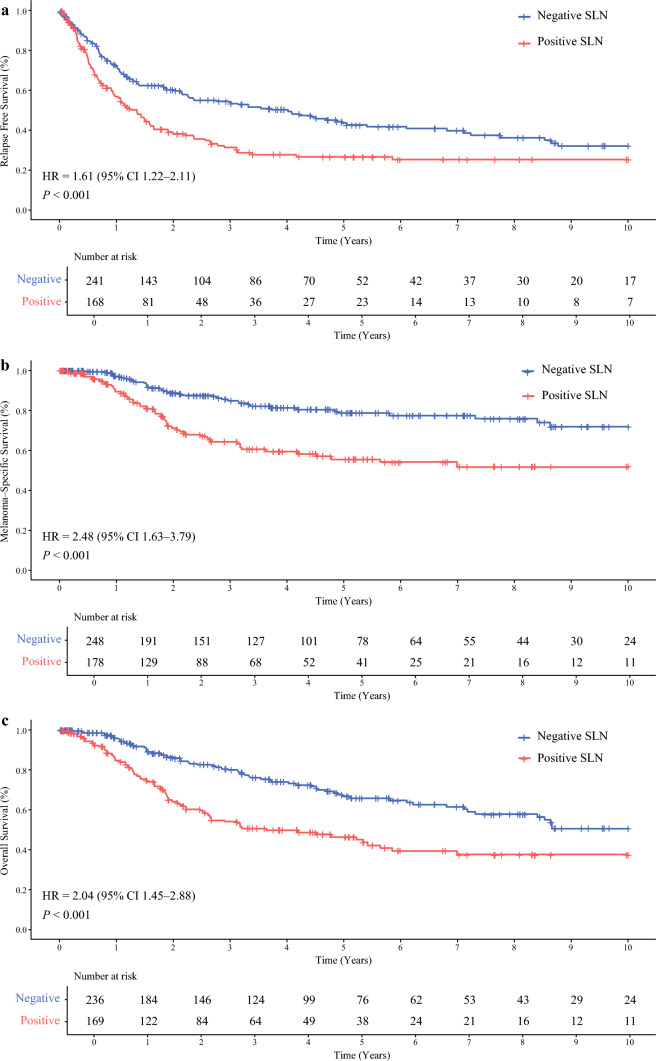
Kaplan–Meier curves for relapse-free survival **A**, melanoma-specific survival **B**, and overall survival **C** showing the prognostic value of SLN status in a subgroup of patients with non-nodal regional metastases. Curves are derived from the imputed dataset for visualization, and the HR and 95% CI are reported from pooled Cox proportional hazards models across multiple imputations (Rubin’s rule); *P*-values are pooled log-rank tests across imputations (Fisher’s method); *SLN* sentinel lymph node, *HR* hazard ratio, *CI* confidence interval

**Fig. 3:**
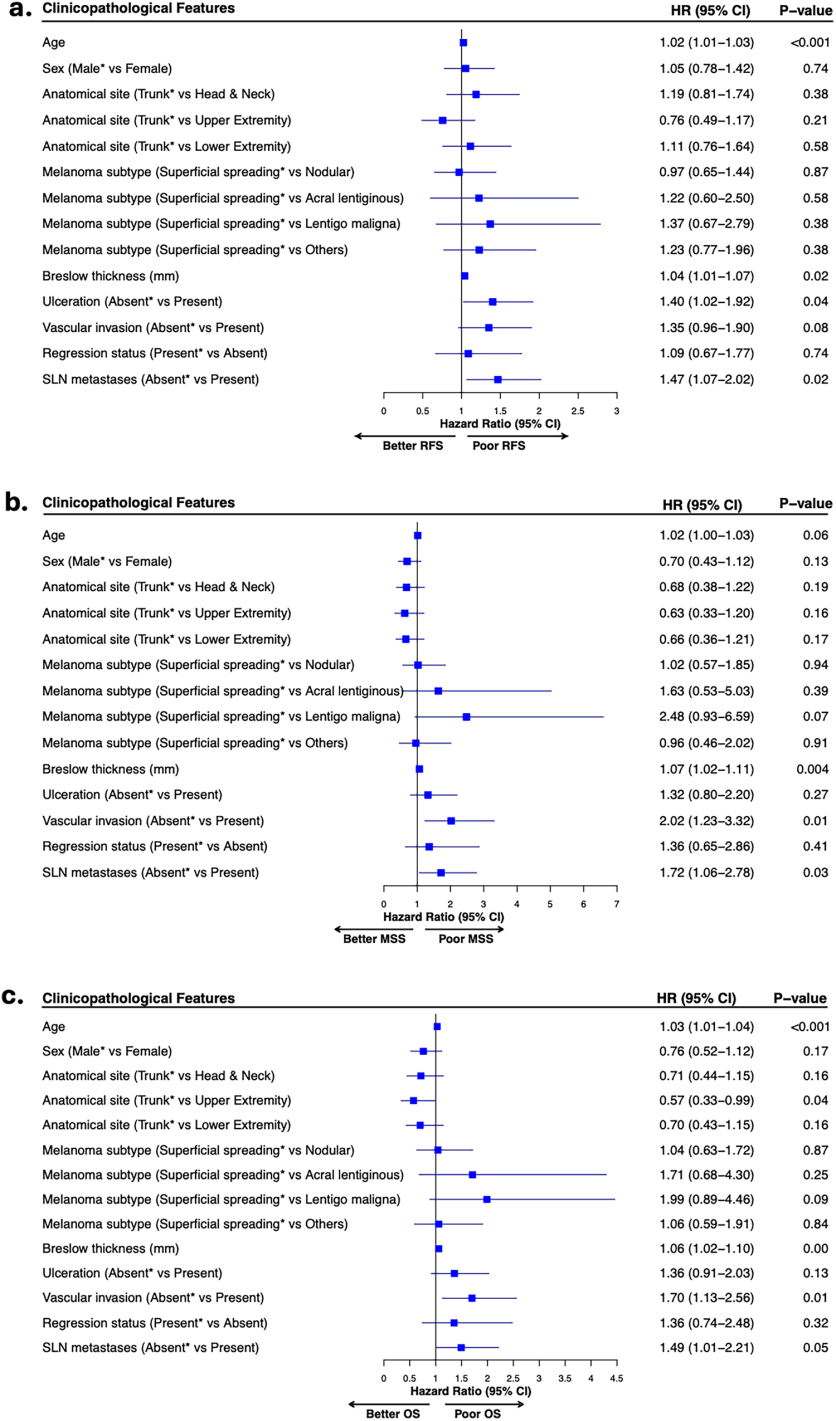
Forest plots derived from multivariable Cox regression analyses showing the adjusted hazard ratios for clinicopathological features associated with relapse-free survival, *n* = 409 **A**, melanoma-specific survival, *n* = 426 **B**, and overall survival, *n* = 405 **C** in a subgroup of patients with non-nodal regional metastasis; age and Breslow thickness are used as continuous variables; *indicates reference categories; *HR* hazard ratio, *CI* confidence intervals, *RFS* relapse-free survival, *MSS* melanoma-specific survival, *OS* overall survival

The results of the univariate analysis stratified by SLN status showed that SLN positivity was associated with significantly worse prognosis in the subgroup of patients with de novo non-nodal regional metastasis (RFS: HR 1.61; 95% CI 1.22–2.11, *P* < 0.001; MSS: HR 2.48; 95% CI 1.63–3.79,* P* < 0.001, and OS; HR 2.04; 95% CI 1.45–2.88, *P* < 0.001) (Fig. 2). The higher hazards associated with positive SLN status were maintained in the subsequent multivariable analyses for RFS (HR 1.47; 95% CI 1.07–2.02, *P* = 0.02), MSS (HR 1.72; 95% CI 1.06–2.78, *P* = 0.03), and OS (HR 1.49; 95% CI 1.01–2.21, *P* = 0.05) (Fig. 3). We did not find statistically significant prognostic associations when microsatellites, satellites, and in-transit metastases were examined individually in the full cohort or in the SLN-positive subgroup (data not shown).

### Prognostic Value of Non-nodal Regional Metastasis in Patients with a Negative Sentinel Lymph Node

Patients presenting with de novo non-nodal regional metastases but a negative SLN are an uncommon and poorly characterized subgroup. We next investigated whether the presence of non-nodal regional metastases provides prognostic value in patients with negative SLNs. In the SLNWG cohort, a negative SLN status was recorded in 84.4% (*n* = 10,671) of patients. Among these, non-nodal regional metastases were identified in 2% (*n* = 227) of the patients.

Cumulative incidence analyses demonstrated that patients with negative SLN status and non-nodal regional metastases had a significantly higher risk of local (HR 4.37; 95% CI 2.84–6.74, *P* < 0.001), regional (HR 3.90; 95% CI 2.83–5.37, *P* < 0.001), and distant relapse (HR 1.86; 95% CI 1.24–2.79, *P* = 0.003) compared with those without non-nodal regional metastases (Supplementary Fig. 1).

**Fig. 4:**
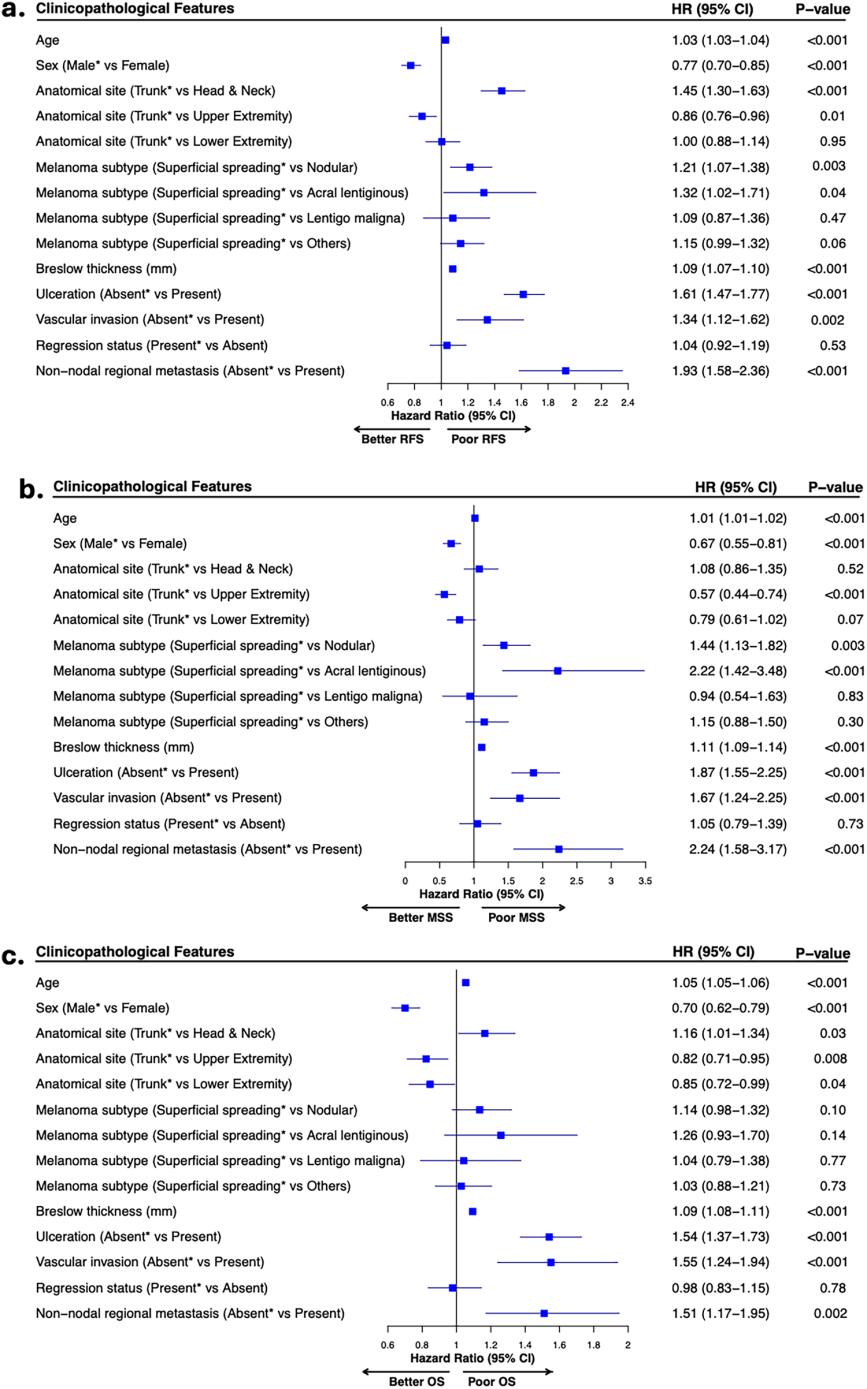
Forest plots derived from multivariable Cox regression analyses showing the adjusted hazard ratios for clinicopathological features associated with relapse-free survival, *n* = 409 **A**, melanoma-specific survival, *n* = 426 **B**, and overall survival, *n* = 405 **C** in a subgroup of patients with negative sentinel lymph node status; age and Breslow thickness are used as continuous variables; *indicates reference categories; *HR* hazard ratio, *CI* confidence intervals, *RFS* relapse-free survival, *MSS* melanoma-specific survival, *OS* overall survival

Univariable analysis showed that the presence of non-nodal regional metastases was associated with significantly poor RFS, MSS, and OS (pooled log-rank *P* < 0.001) (Supplementary Fig. 2). Importantly, this adverse prognostic effect persisted in the multivariable model after adjusting for established clinicopathological factors, confirming that de novo non-nodal regional metastases are an independent prognostic factor associated with poor RFS (HR 1.93; 95% CI 1.58–2.36, *P* < 0.001), MSS (HR 2.24; 95% CI 1.58–3.17, *P* < 0.001), and OS (HR 1.51; 95% CI 1.17–1.95, *P* = 0.002) (Fig. 4).

Overall, these results demonstrate that despite negative SLN status, these patients have poor outcomes, suggesting that non-nodal regional metastases represent a distinct adverse prognostic factor that overrides the protective effect of a negative SLN.

## Discussion

The salient results of this retrospective analysis of the SLNWG database, comprising more than 13,000 patients with cutaneous melanoma, can be summarized as follows: (a) the presence of non-nodal regional metastases is an independent factor associated with SLN positivity; (b) SLN positivity is associated with adverse prognosis in patients with non-nodal regional metastases; and (c) even in the subset of patients with negative SLN status, the presence of non-nodal regional metastases confers a poor prognosis, highlighting their independent contribution to the risk. These findings, while consistent with prior reports,^4,9,10,16,19^ add further evidence from a large, well-annotated, multi-institutional cohort.

The AJCC 8th edition classification assigns patients with non-nodal regional metastases to N1c, N2c, or N3c categories, depending on the presence of concurrent regional nodal involvement. This results in patients being assigned to different stage III subgroups (e.g., IIIB, III C, or IIID). While this framework implies that SLN positivity provides prognostic separation, contemporary evidence is limited regarding whether SLN status meaningfully alters outcomes once non-nodal regional metastases are present or whether the prognosis is instead driven predominantly by the adverse biology of non-nodal disease. Thus, it remains unclear whether SLN status provides independent prognostic value in this context. Our results demonstrate that non-nodal regional metastases provide independent prognostic information, with adverse outcomes observed even among patients with negative SLNs. These findings provide real-world validation of the AJCC framework and support the continued clinical relevance of SLN status in this rare but high-risk population of patients only when they are clinically node negative. Recognizing that these patients are already stage III, SLN status might not influence neoadjuvant therapy eligibility, but it provides additional prognostic refinement when positive. It should be acknowledged that SLNB could provide additional nodal basin control, especially in patients who are ineligible for neoadjuvant treatments or adjuvant treatments.^20^

The adverse prognosis observed in patients with non-nodal regional metastases, even when the SLN is negative, may reflect several mechanisms. In cutaneous melanoma, skip metastases are well described, whereby tumor cells bypass the sentinel node and disseminate via alternative lymphatic pathways.^21^ In addition, micrometastatic disease within the SLN may remain undetected, leading to a false negative SLN status.^22–24^ The variability of the cutaneous lymphatic drainage, though rare, could also account for regional tumor spread without SLN involvement.^25^ Finally, the presence of non-nodal regional metastases may represent an inherently aggressive tumor phenotype with metastatic potential that operates independently of the nodal status. From a clinical perspective, these results reinforce that patients with non-nodal regional metastases constitute a high-risk group warranting intensified surveillance, tailored counselling, and adjuvant and neoadjuvant therapies regardless of the SLN status.

The role of SLN biopsy in patients with de novo in-transit metastases remains unclear, a view reflected in prior reports noting the intrinsically high risk of subsequent distant spread and the limited likelihood that occult nodal staging could alter systemic therapy decisions; consequently, the value of SLN biopsy in this group has been questionable and is not a routinely performed procedure across centers.^26,27^ However, contemporary evidence suggests that SLN is still prognostically informative in clinically node-negative patients who present with in-transit disease, where it reveals occult nodal involvement in approximately 13–36% of patients.^28,29^ Moreover, the outcomes of patients with in-transit disease are sharply stratified by the overall regional nodal involvement, regardless of whether detected clinically or by pathological assessment (including SLN biopsy), with poor survival in those with nodal involvement.^6,28^ Given the expanded use of adjuvant anti-PD1 and BRAF/MEK therapy in resectable stage III/IV melanoma, precise nodal staging is increasingly consequential. SLN-defined nodal status may help to identify the high-risk subset among those with non-nodal regional metastases most likely to be considered for adjuvant therapy or trial enrollment in the context of de novo in-transit metastases.^30–32^

Our findings confirm that de novo non-nodal regional metastases represent a biologically aggressive phenotype. These lesions were strongly associated with SLN positivity, and SLN status remained an independent predictor of survival within this subgroup. Importantly, the presence of non-nodal regional metastases conferred poor outcomes even among patients with negative SLNs, indicating that this adverse biology partially overrides the protective effect typically associated with SLN negativity.

In contemporary practice, patients with clinically evident stage III disease are generally managed with neoadjuvant systemic therapy. Furthermore, SLN status does not determine therapy eligibility in most patients with overt non-nodal regional metastases. However, our results show that SLN status continues to provide incremental prognostic information, identifying a subset with markedly worse outcomes.

Given the rarity of de novo non-nodal regional metastases and the contemporary preference for neoadjuvant therapy in stage III melanoma, SLN biopsy has only a small routine role in this patient population, except in rare node-negative patients who may have a contraindication to neoadjuvant therapy, or when a clinically suspicious satellite or in-transit lesion requires histologic confirmation before assigning stage III status. Overall, while SLN status offers incremental prognostic insight, it might not have a huge practical influence on systemic therapy decisions in patients with clinically evident stage III disease.

The strengths of our study include a large, multi-institutional, well-characterized cohort. While most prior studies have evaluated microsatellites, satellites, and in-transit metastases separately in relation to SLN status and prognosis, we analyzed them collectively as non-nodal regional metastases, consistent with the AJCC classification. Nonetheless, we acknowledge that these are relatively rare entities and grouping them may obscure potential biological or prognostic differences among subtypes. However, this approach increased the subgroup sample size, thereby enhancing the robustness and stability of the statistical comparisons. Another strength of this study is the use of multiple imputations by chained equations to handle the missing data, which preserved the full sample size and reduced the potential bias compared with complete-case analyses.^33^ However, this method relies on the assumption that data were missing at random, which cannot be definitively verified.

This study has several limitations worthy of acknowledgement. The retrospective nature of the study cohort introduces the potential for selection bias and heterogeneity in clinical practice. Non-nodal regional metastases remain a rare clinical entity, limiting meaningful subgroup analyses despite the overall large cohort size. Additionally, the median follow-up of the SLNWG database is relatively short, 3.2 years, limiting the assessment of late events; as such, survival analyses are primarily driven by early events, and long-term associations should be interpreted with caution. This study spans a long inclusion period (1988–2025), during which effective systemic therapies became available. As a result, the prognostic effect of nodal status on MSS may have been attenuated, particularly among patients who received adjuvant therapies, since node-positive patients could have benefited from it.^31^ However, treatment data were largely missing across contributing centers, precluding a robust investigation of therapy-related outcomes. Although the SLNWG registry distinguishes clinically evident in-transit metastases and satellites from pathologically detected microsatellites, it does not capture information on whether satellites or in-transit metastases were identified on the initial diagnostic biopsy or subsequent WLE. Consequently, the precise point of detection of microsatellites cannot be determined, limiting assessment of how these findings may have influenced SLNB decision-making. Finally, the AJCC classification of non-nodal regional metastases was refined in the 8th edition,^1^ which may have influenced case ascertainment and contributed to heterogeneity in observed prognostic associations.

In summary, our results demonstrate that non-nodal regional metastases are independently associated with SLN positivity and poorer prognosis in patients with cutaneous melanoma, both in those with positive and negative SLNs. These findings reinforce that the aggressive biology of non-nodal regional metastases ought to be considered in ongoing efforts to refine risk stratification and treatment strategies for this rare clinical entity.

## Supplementary Information

Below is the link to the electronic supplementary material.

Supplementary file1 (DOCX 2784 KB)
