# Publisher Correction: Prognostic Value of Non-nodal Regional Metastases in Predicting Sentinel Lymph Node Status in Cutaneous Melanoma: Multicenter Analysis of the Sentinel Lymph Node Working Group Database

**DOI:** 10.1245/s10434-026-19230-y

**Published:** 2026-02-16

**Authors:** Nazia Riaz, Stanley P. Leong, Mohammed Kashani-Sabet, Richard L. White, John T. Vetto, Schlomo Schneebaum, Cristina O’Donoghue, J. Harrison Howard, Eli Avisar, Jukes P. Namm, Heidi Kosiorek, Barbara Pockaj, Mark Faries, Giorgos Karakousis, Jonathan S. Zager, Roger Olofsson Bagge

**Affiliations:** 1 Department of Surgery, Institute of Clinical Sciences, Sahlgrenska Academy, University of Gothenburg, Gothenburg, Sweden; 2 Department of Surgery, Sahlgrenska University Hospital, Gothenburg, Västra Götalandsregionen Sweden; 3 Center for Melanoma Research and Treatment, California Pacific Medical Center and Research Institute, San Francisco, CA USA; 4 Department of Surgery, Atrium Health Levine Cancer Center, Charlotte, NC USA; 5 Department of Surgery and Division of Surgical Oncology, Oregon Health & Science University, Portland, OR USA; 6 Department of Surgery, Tel Aviv University, Tel Aviv, Israel; 7 Department of Surgery, Rush University Medical Center, Chicago, IL USA; 8 Department of Surgery, University of South Alabama, Mobile, AL USA; 9 Department of Surgical Oncology, University of Miami School of Medicine, Miami, FL USA; 10 Department of Surgery, Loma Linda University, Loma Linda, CA USA; 11 Department of Quantitative Health Sciences, Mayo Clinic, Scottsdale, AR USA; 12 Department of General Surgery, Division of Surgical Oncology, Mayo Clinic Arizona, Phoenix, AR USA; 13 Department of Surgery, Angeles Clinic and Research Institute, Cedars-Sinai Medical Center, Los Angeles, CA USA; 14 Division of Endocrine and Oncologic Surgery, University of Pennsylvania School of Medicine, Philadelphia, PA USA; 15 Departments of Cutaneous Oncology and Sarcoma, Moffit Cancer Center, Tampa, FL USA

**Correction to: Annals of Surgical Oncology** 10.1245/s10434-026-19086-2

In the original online version of this article there were errors in Figs. 1, 3, and 4. The original article was corrected.

