# Supplementary material for: Prognostic Value of Non-nodal Regional Metastases in Predicting Sentinel Lymph Node Status in Cutaneous Melanoma: Multicenter Analysis of the Sentinel Lymph Node Working Group Database
Source: Ann Surg Oncol. 2026 Jan 23;33(5):4011–22. doi: 10.1245/s10434-026-19086-2 (PMC13083337; doi:10.1245/s10434-026-19086-2)
Supplement: Supplementary file 1 — Supplementary file1 (DOCX 2784 KB) [file 10434_2026_19086_MOESM1_ESM.docx]

**Supplementary data**

**a.**

**b.**

**c.**

**Figure S1:** Cumulative incidence of (A) local, (B) regional, and (C) distant relapses in patients with negative SLN status in the SLNWG cohort (n=10643) stratified by non-nodal regional recurrences (absent vs present). Analyses used a competing-risks framework: relapse types other than the ones shown in each panel were modelled as competing events (not censored), so patients with a non-target relapse were not counted as the risk for the target relapse in that panel. The cumulative incidence functions shown are based on descriptive analyses (complete-case, no imputation)

Abbreviations: HR, hazard ratio; CI, confidence intervals

**b.**

**a.**

**c.**

**Figure S2:** Kaplan Meier curves for relapse-free survival (A), melanoma-specific survival (B), and overall survival (C) showing the prognostic value of non-nodal regional metastases (absent vs present) in a subgroup of patients with negative SLN status. Curves are derived from the imputed dataset for visualization, and the HR and 95% CI are reported from pooled Cox proportional hazards models across multiple imputations (Rubin’s rule). *P* values are pooled log-rank tests across imputations (Fisher’s method).

Abbreviations: HR, hazard ratio; CI, confidence intervals
